# Shaping oral and intestinal microbiota and the immune system during the first 1,000 days of life

**DOI:** 10.3389/fped.2025.1471743

**Published:** 2025-01-21

**Authors:** Jie Zhu, Mingxin He, Simin Li, Yumeng Lei, Xiaochen Xiang, Zhi Guo, Qiang Wang

**Affiliations:** ^1^ Institute of Infection, Immunology and Tumor Microenvironment, Wuchang Hospital Affiliated to Wuhan University of Science and Technology, Medical College, Wuhan University of Science and Technology, Wuhan, China; ^2^ Department of Hematology, Huazhong University of Science and Technology Union Shenzhen Hospital, Shenzhen, China

**Keywords:** first 1,000 days of life, oral microbiota, intestinal microbiota, microecology, immune system

## Abstract

The first 1, 000 days of life, from the fetal stage of a woman's pregnancy to 2 years of age after the baby is born, is a critical period for microbial colonization of the body and development of the immune system. The immune system and microbiota exhibit great plasticity at this stage and play a crucial role in subsequent development and future health. Two-way communication and interaction between immune system and microbiota is helpful to maintain human microecological balance and immune homeostasis. Currently, there is a growing interest in the important role of the microbiota in the newborn, and it is believed that the absence or dysbiosis of human commensal microbiota early in life can have lasting health consequences. Thus, this paper summarizes research advances in the establishment of the oral and intestinal microbiome and immune system in early life, emphasizing the substantial impact of microbiota diversity in the prenatal and early postnatal periods, and summarizes that maternal microbes, mode of delivery, feeding practices, antibiotics, probiotics, and the environment shape the oral and intestinal microbiota of infants in the first 1, 000 days of life and their association with the immune system.

## 1 Introduction

The immune system in early life does not develop in isolation, but is strongly influenced by maternal and autoantigens, commensal bacteria, and pathogens (1). The microbiota establishes or maintains homeostasis when the balance of immune system tolerance to commensal, conditionally pathogenic and harmful bacteria is established (2); microbiota regulate the function and activity of immune cells, stimulate mucosal specificity and activate the immune system and induce the development and maturation of immune cells and organs, with health-promoting and beneficial immunomodulatory properties (3). Therefore, the two-way communication between immune system and microbiota is helpful to maintain the microecological balance and immune homeostasis of human body. Indeed, microbe-dependent modulation of the host immune system has a limited window of opportunity, and there is no way to make up for the missed window early in life in adulthood, which may even promote inflammation or increase the risk of disease progression, making it particularly important to focus on the establishment of the microbiota and the immune system early in life.

**Figure 1:**
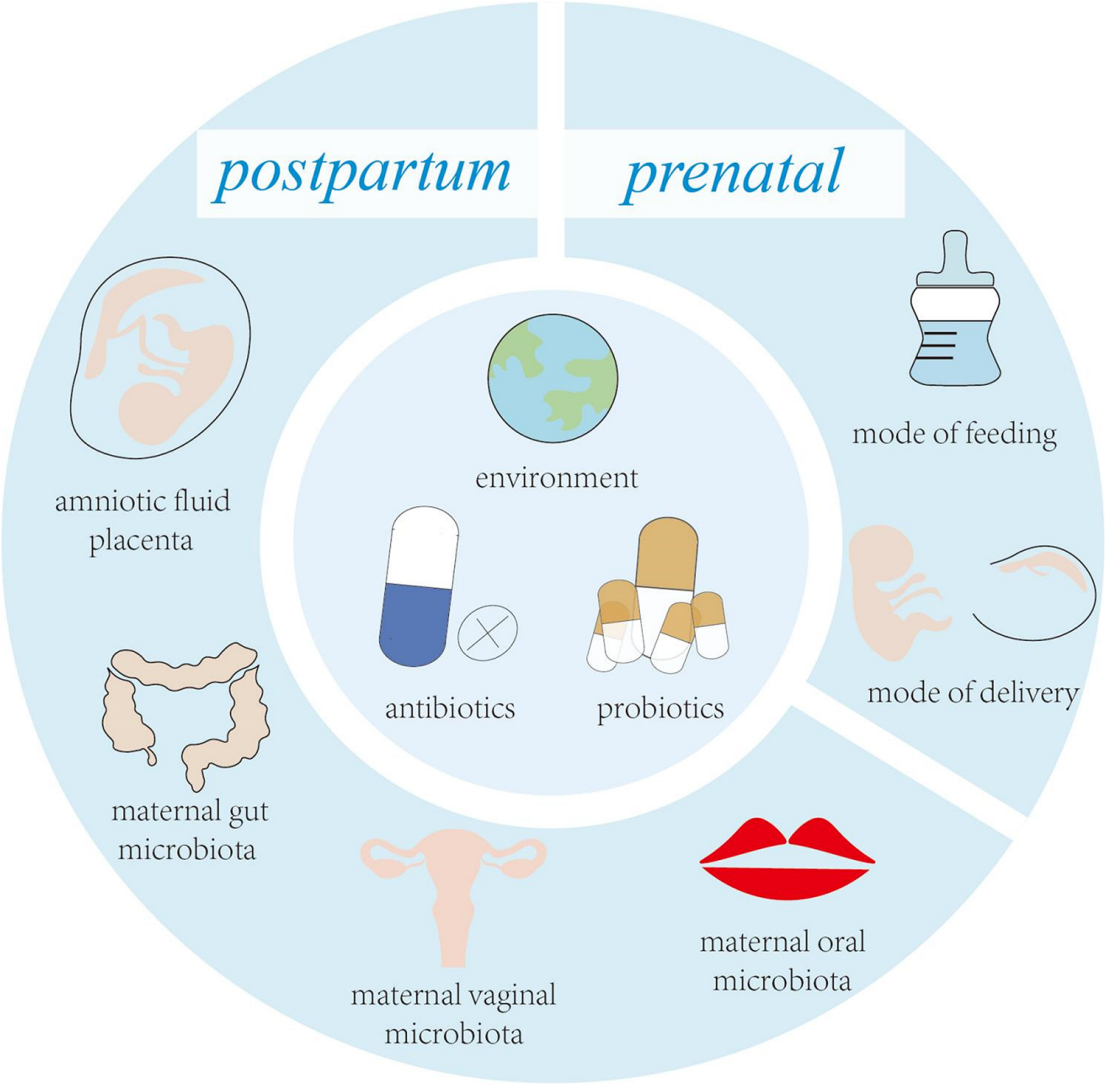
Key factors influencing early colonizers before and after birth.

*Streptococcus spp.* are the first bacteria to colonize the infant's mouth and are known as “early colonizers” (4). Early colonization affects subsequent microbial colonization due to the occupation of binding sites, efficient use of nutrients, production of antimicrobial agents and end products, and changes in the environment (5). Therefore, early colonization is very important for the development of microbiota, and may have long-term consequences (6). Facultative anaerobic bacteria are early colonizers in the human intestinal and are distinctly dominant in the first few weeks of life, such as *Enterobacterales*, *Enterococci* and *Staphylococci*. Subsequently, the dominant microbiota gradually changed to anaerobic bacteria, such as *C. leptum subgroup*, *Bifidobacterium* and *B. fragilis* (7). With the introduction of solid food, intestinal microbiota are dominated by *Firmicutes* and *Bacteroidetes* (8). Acquisition of early colonizers may be altered by a variety of maternal and infant factors (Figure 1), which may lead to differences in oral and intestinal microbiota development (9, 10).

Disruption of early oral colonization may affect the progression of oral and systemic disease in children. The oral microbiota has shown significant correlations with systemic disorders such as weight gain, rheumatoid arthritis (RA), and autism (11–13). In addition, the oral cavity is the window to the gut, and the oral microbiota affects the colonization of the gut microbiota to a certain extent, and it has been shown that the oral microbiota shows its relevance and possibilities as a substitute for the gut microbiota (14). Intestinal microbiota have the ability to generate resistance to pathogens and enhance the resistance of the immune system through colonization of mucosa surface and production of different antimicrobial substances, which play an important role in maintaining normal gut physiology and health. Thus, the oral and intestinal microbiota has a unique role in promoting immune system development and modulating host defence (15, 16). The aim of this review is to provide a summary of the latest research advances in the establishment of the oral and gut microbiome and the immune system during the first 1,000 days of life. At the same time, it is emphasized that maternal factors, mode of delivery, breastfeeding, antibiotics, host factors, and living environment reciprocally shape the oral and intestinal microbiota of infants during the first 1,000 days of life and their association with the immune system.

## 2 Prenatal factors affecting the development of the fetal microbiota and immune system

### 2.1 Effects of maternal microbiota on the fetus

For many years, scientists and doctors have believed that the fetus grows and develops in a sterile environment. Recent research data questioned this common understanding. New research found that there are microbiota in meconium, placenta and amniotic fluid, and put forward the view that uterus is not a sterile organ (17). The most common bacteria in meconium samples are Staphylococcus, followed by *Enterobacteriaceae*, *Enterococcus*, *Lactobacillus* and *Bifidobacterium* (18–20). However, Rehbinder et al. suggested that the presence of foetal faecal microbiota was the result of DNA contamination from laboratory reagents or acquired during labour (21). Stinson et al. combined PacBio single-molecule real-time (SMRT) sequencing technology with a workflow designed to reduce contamination and showed that the foetal faecal and amniotic fluid microbiomes were present beyond background contamination levels (22). Meanwhile, Aagaard et al. performed the first macrogenomic characterisation of the placental microbiome using whole-genome birdshot sequencing and 454 pyrophosphate sequencing, and found that the placenta contains a unique microbiome composed of a non-pathogenic gut microbiota dominated by *Proteobacteria*, and composed of non-pathogenic intestinal microbiota of *Firmicutes*, *Bacteroidetes* and *Fusobacteria phyla*. At the same time, it is proposed that placental microorganisms are most similar to maternal oral microorganisms and that their composition may also influence pregnancy outcome (23). The microbiota in amniotic fluid is similar to that in placenta, and an imbalance in its microbial composition may lead to chorioamnionitis. Previous studies have shown that the microbial status in amniotic fluid can be used to predict the occurrence of premature birth (24). Preterm birth and delivery are associated with bacterial colonization of the amniotic cavity and fetal membranes, and the amount of microbial DNA in amniotic fluid correlates with increased levels of leukocytes and inflammatory mediators, suggesting that a higher microbial load can lead to inflammation, which may be an endocrine mechanism that triggers preterm labor (25). These results suggest that colonization of the fetal oral cavity and intestine by the microbiota may have occurred during pregnancy.

**Figure 2:**
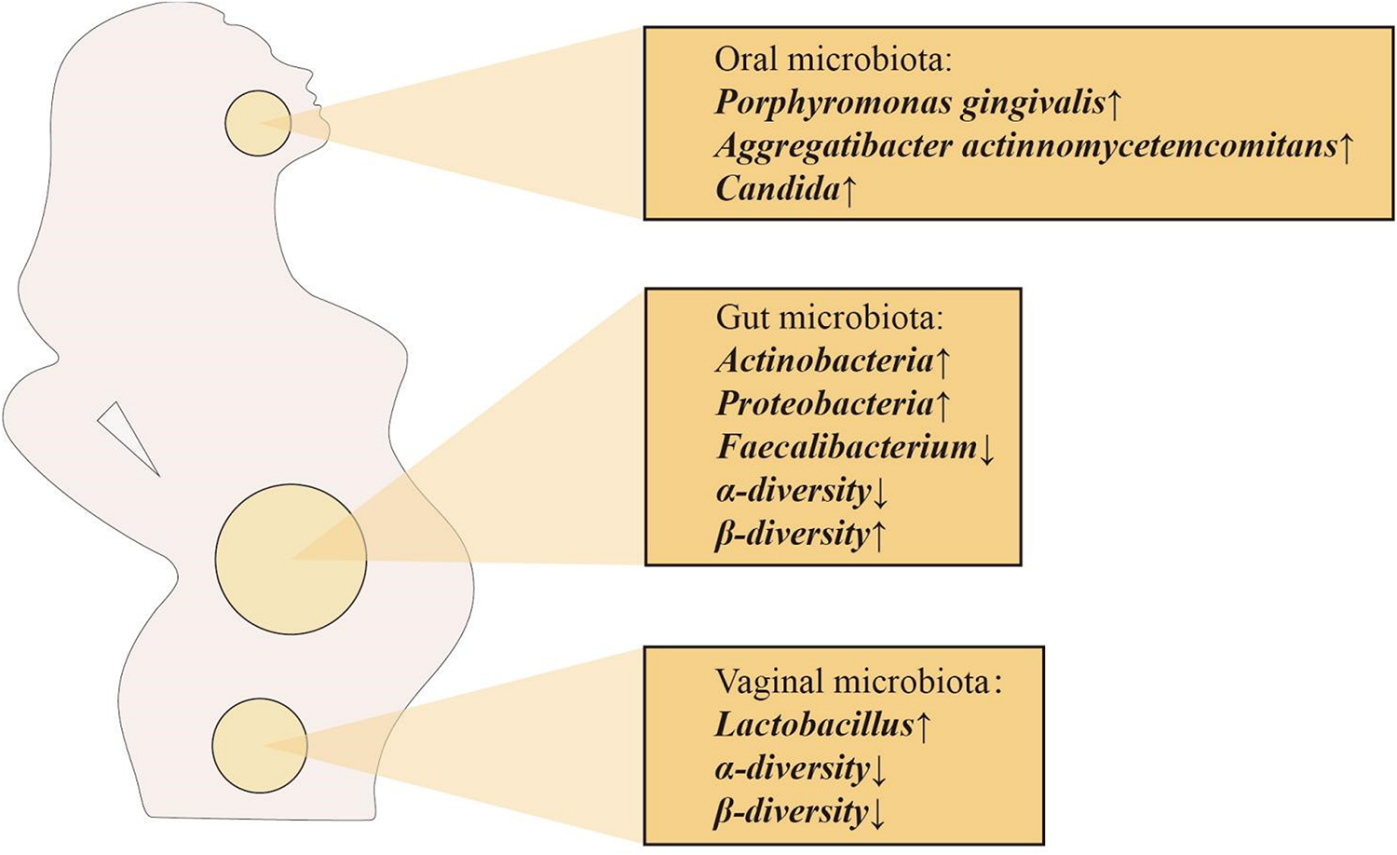
Major changes in the maternal oral, intestinal and vaginal microbiota during pregnancy.

Vertical transfer of bacterial species between mother and baby occurs before the baby is born, suggesting that the physiological state of the mother can influence the microecology of the fetus. Maternal microbiota is a key source of microbiota during initial colonization of the fetal oral and gut microbiota. Maternal factors that shape the fetal microbiota are mainly the maternal oral, intestinal and vaginal microbiota (26). Interestingly, the composition of the maternal oral, intestinal and vaginal microbiota changes dramatically during pregnancy (Figure 2), which may be the source of the close connection between the maternal microbiota and the fetal and infant microbiota. The total number of oral microbiota was higher than that of non-pregnant bacteria at different stages of pregnancy. In the early and second trimester of pregnancy, the abundance of *Porphyromonas gingivalis* and *Aggregatibacter actinomycetemcomitans* increased significantly, *Candida* levels were significantly higher in mid and late pregnancy, and the abundance of *Actinomyces* was even more significantly higher throughout pregnancy, changes that may be due to changes in the overall immune status during pregnancy (27–30). From the first trimester to the third trimester, changes in the maternal gut microbiota are mainly manifested by increased abundance of *Actinomycetes* and *Proteobacteria*, a significant decrease in the levels of butyrate-producing *Faecalibacterium* with anti-inflammatory activity in the third trimester, and a decrease in α-diversity at the individual level. In addition, in the third trimester of pregnancy, the β-diversity of the maternal microbiota increases among individuals, reflecting the immune manifestations of chronic inflammation (29, 31, 32). Alterations in the maternal gut microbiota during pregnancy play a positive role in the regulation of metabolic responses in the fetus (33). In view of the influence of intestinal microbiota on fetal immune response, the adaptation and regulation of maternal immune response during pregnancy is a necessary condition for fetal tissue cells to be accepted by the mother and avoid maternal immune rejection, while maintaining their own immune defense mechanism to ensure the survival of the mother and fetus (34). The vaginal microbiota also changes significantly during pregnancy, with a decrease in overall diversity and number of organisms in the first trimester, but an increase in the stability of the microbiota composition, as well as an enrichment of *Lactobacillus*, which leads to a decrease in vaginal pH and an increase in secretions (35). Studies have shown that the increase of *Lactobacillus* has a direct or indirect protective effect on maintaining the stability of the vaginal microbiota during pregnancy and preventing ascending infections (36). In addition, a decrease in the proportion of *Lactobacillus* is associated with adverse pregnancy outcomes, such as preterm birth, miscarriage, etc (37).

The above changes in the mother's own microbiota during pregnancy can reach the placenta and affect the fetal microbiota through different routes of transmission, with blood transmission being the most direct and effective route. Scientists hypothesize that the pathway of transmission of the microbiota from mother to fetus is similar to the potential pathway for intrauterine infections (38). Some of the maternal immune cells are thought to be transporter cells for placenta-acquired microbiota (39). For example, dendritic cells (DCs) can cross the gap between intestinal epithelial cells and capture bacteria directly from the intestinal lumen. However, DCs do not kill the captured microorganisms, which can be transmitted through the dendritic cells through the bloodstream to other sites, such as the placenta and amniotic fluid, and through the umbilical cord to the fetus (40). Intrauterine microbiota colonization may occur via the ascending route through the genitourinary tracts such as the urinary tract, cervix, and vagina, as well as the hematogenous route through the placenta after translocation from the digestive tracts such as the oral cavity and intestines (41). Microbiota associated with the gastrointestinal tract such as *Bacteroides* and *Proteus* as well as microbiota associated with the vaginal microbiota such as *Lactobacillus iners*, *L. crispatus*, and *Prevotella amnii* were found in endometrial samples, so Verstraelen et al. concluded that the endometrium is colonized by both vaginal and intestinal microbiota (42). In addition, transplacental passage of these colonized microorganisms or their metabolites may result in increased expression of microbe-associated molecular pattern (MAMP) recognizing receptors, thymic regulatory T (Treg) cell promotion, and the establishment of dendritic cell (DC) networks in the gut, lung, and other tissues, as well as accelerated transitions from TH2 to TH1, interferon-regulated transcription factor 7 (RF7), and TH-17 (43).

### 2.2 Effects of antenatal antibiotic use on the fetus

Current clinical evidence suggests that antibiotics used by pregnant women may cross the placental barrier and reach the fetus, directly or indirectly disrupting the oral and intestinal microbiota of the fetus (Table 1) (48). By comparing fecal samples from PAT (prenatal antibiotic treatment) and PAF (prenatal antibiotic-free) preterm infants on days 7 and 14 after birth, Zou et al. found that prenatal antibiotic exposure resulted in a decrease in the intestinal microbiota of preterm infants in Bacteroides spp. (PAF 9.11%, PAT 2.93%) and an increase in Escherichia-Shigella (d7, PAF 27.35%, PAT 43.35%; d14, PAF 29.47%, PAT 40.36%) increases (49). A meta-analysis showed a significant decrease in the relative abundance of Actinobacteria and an increase in the abundance of Firmicutes and Proteobacteria in infants whose mothers were treated with antibiotics before or during labor and delivery, compared to infants who were not exposed to antibiotics (50). In the current study, Marilen et al. used a mouse pregnancy model to explore the effects of antibiotic treatment on maternal immunity, and the microbial intervention strategy chosen was shown to affect offspring immunity (51). Using a mouse model, Xu et al. showed that prenatal antibiotic exposure led to a reduction in microbiota-derived butyrate production, which in turn enhanced neonatal ILC2 activation by downregulating IFN1 signaling (52). At the same time, antibiotic use in pregnant and lactating females reduces antiviral-specific immune responses in pups, suggesting that antibiotics cause extensive immune damage in the offspring (53). A large number of studies have shown that maternal antibiotic use increases the risk of obesity, otitis media, asthma and other diseases after birth (54–56). The above results suggest that prenatal antibiotic use can affect the baby's oral and intestinal microbiota, which in turn affects the development of the baby's immune system.

**Table 1:** Effects of maternal prenatal or perinatal antibiotic use on the infant microbiota.

Country	Types of research	Sample size	Microbiota changes	Reference
Canada	Longitudinal study, single-center	198 infants	IAP for maternal vaginal delivery was associated with decreased infant gut microbiota richness at 3 months (*P* = 0.005), whereas IAP for emergency CS was associated with increased microbiota diversity at 1 year of age (*P* < 0.001), *Bacteroides* and *Parabacteroides* were underrepresented, while *Enterococcus* and *Clostridium* were overrepresented	(44)
Suomi	Control study, single-center	149 infants	Compared to unexposed infants, infants with IAP had decreased abundance of *Bacteroidetes* (*P* = 0.04) and increased abundance of *Firmicutes* (*P* = 0.048) especially the *Staphylococcus* and *Clostridium genera*	(45)
America	Longitudinal study, single-center	266 infants	There was a significant difference in infant microbial diversity in IAP infants compared to those in the no-antibiotic group at 6 weeks (Shannon t statistic = −2.374, adjusted *P* = 0.02; Simpson t statistic = −1.907, adjusted *P* = 0.06), with a decrease in the relative abundance of *Bacteroides*, *Bifidobacterium* and *Blautia* adjusted *P* = 0.06), with a decrease in the relative abundance of Bacteroides, Bifidobacterium and Blautia	(46)
Spain	Longitudinal study, single-center	40 infants	The establishment pattern of the gut microbiota of IAP infants changed, with lower relative proportions of *Bifidobacteriaceae* and unclassified *Actinobacteria* (*p* < 0.05)	(47)

### 2.3 Effects of prenatal environmental exposure on the fetus

Exposure to environmental pollutants and bacteria early in life can affect the establishment of the fetal oral and intestinal microbiota and the development of the immune system. Some recent studies have shown that maternal inhalation of PM2.5 may induce oxidative stress, inflammatory responses, endocrine disruption and epigenetic changes, thus indirectly affecting normal fetal development (52). Inhaled PM2.5 may also penetrate the alveolar epithelial barrier and subsequently enter the circulation and be deposited in the placenta, where these particles may directly damage the structure and function of the placenta and further affect fetal growth (57). Tao et al. exposed pregnant mice to filtered air (FA) or concentrated ambient PM2.5 (CAP) and found that CAP exposure altered the metabolome and disrupted metabolic pathways (e.g., amino acid, lipid pathways) in maternal serum and placenta (58). Prenatal maternal exposure to air pollutants, especially in the first and third trimesters, affects the distribution of white blood cells in the fetus, and may also lead to an imbalance in fetal T helper(Th) cell subsets, increasing the risk of allergic reactions in children (59, 60). Loss et al. assessed mRNA expression of Toll-like receptors (TLR) 1 through TLR9 and CD14 in cord blood and found that gene expression of innate immunity receptors was higher overall in the cord blood of newborns from a rural setting (*P* for multifactorial multivariate ANOVA = .041), particularly so for TLR7 [adjusted geometric means ratio (aGMR), 1.15; 95% CI, 1.02–1.30] and TLR8 (aGMR, 1.15; 95% CI, 1.04–1.26) (61). In addition, prenatal exposure to farms can modulate the immune system of offspring, and from umbilical cord blood cells obtained from mothers born to mothers who were exposed to farms before birth, a decreased TH2 immune response, a decrease in the number of white blood cells, an increase in the number of Treg cells, and an increase in immunosuppressive ability, while pro-inflammatory cytokines such as TNF-α and IL-6 in umbilical cord blood are increased (62, 63).

## 3 Postnatal factors affecting the development of the infant microbiota and immune system

### 3.1 Effects of mode of delivery on infants

Although the fetus has begun to colonize and establish microbiota in the mother's body, the main maternal origin of microbiota may begin to build up in large quantities during production. Vaginal delivery(VD) or caesarean section(CS) largely determines the commensal microbiota that begins to settle in the newborn, and there are significant differences in the degree of oral and intestinal microbial development between different modes of delivery (64, 65).

VD infants have oral and intestinal microbiota enriched with the mother's vaginal microbiota, such as *Lactobacillus*, *Prevotella* and *Bifidobacterium*, during the first week of life (66). At the same time, VD infants are consistently enriched with maternal gut microbiota such as *Bacteroidetes*, *Bifidobacterium* and *Escherichia coli*, which may be due to the better adaptation of maternally transmitted high-abundance bacteria to the intestinal environment (29). It was found that neonates VD had high levels of gram-negative bacteria, which led to a significant enrichment in lipopolysaccharide (LPS) biosynthesis, and that LPS stimulation of primary human immune cells led to higher levels of TNF-α and IL-18, suggesting a link between the immunostimulatory potential of gut microbial LPS and the overall immune status of neonates (67). Another study also showed that LPS was involved in building tolerance to the colonized gut microbiota and initiating the neonatal immune system, and that stimulatory LPS helped reduce the risk of immune-mediated disease (68). Cord blood from vaginally delivered infants has higher immune cell counts and activity (e.g., neutrophils, monocytes, NK cells), higher concentrations of cytokines and expression levels of TLR 2 and TLR 4, and expression levels of CD 16 and CD 56 surface receptors (68).

Newborns delivered by CS have microbiota similar to the maternal skin microbiota, such as *Staphylococcus*, *Corynebacterium*, *Propionibacterium* and environmental microbiota, whereas *Bifidobacterium spp*. which are common in VD are not found (69). *Bacteroides* are associated with a lower proportion of the oral and intestinal microbiota of neonates born by CS compared with VD, which may further contribute to reduced bacterial diversity in caesarean section infants (70). Compared with VD, the potential pathogens of CS infants are more abundant, such as *Clostridium perfringens* or *Escherichia coli* (71, 72). It may be related to the reduction of leukocytes such as lymphocytes and dendritic cells(DC) in the umbilical cord blood of infants born by CS, as well as the expression of lower levels of the surface innate antigen receptors TLR2 and TLR4 by mononuclear cells in the umbilical cord blood (73, 74). In addition, CS infants had reduced responses to TLR1/2 stimulation of TNF-α and IL-6 (74). CS may affect the critical window period of immune system startup and destroy the mother-to-child transmission and immune stimulation potential of specific microbiota (67). Based on mouse models, it has been found that differences in the earliest initiation of the immune system may continue to affect the development and healthy development of the human immune system (75). Another study also found that newborns born by CS were at higher risk of developing chronic diseases in the future due to changes in early immune system stimulation (76). Compared with VD, CS is associated with an increased risk of childhood immune diseases such as asthma and allergies, leukemia and IBD. The above results suggest that the mode of delivery affects the colonization of neonatal microbiota and the development of the immune system, thereby providing different defenses against infection in the later stages. CS will increase the risk of antibiotic exposure and change the composition of breast milk. At the same time, it leads to an increase in the relative abundance of opportunistic pathogens in neonates, affecting the immune and metabolic development of infants. In addition, by analysing the early microbiota composition and dynamics of 34 mother-infant pairs, Selma-Royo et al. found a significant effect of home vs. hospital births, which was also evident at 6 months of age, but by which time the differences associated with mode of delivery had disappeared (77). Therefore, while focusing on the mode of delivery, relevant factors (e.g., place of delivery, etc.) should be added to the mix to better understand infant bacterial colonisation and its potential long-term impact on infant development.

### 3.2 Effects of feeding methods on infants

Traditionally, breast milk has been considered sterile, but several studies have confirmed that breast milk provides an important source of bacteria for infants, with *Staphylococcus*, *Streptococcus*, *Bifidobacterium*, *Propionibacterium* and *Lactobacillus* present in breast milk samples (78, 79). According to WHO's recommendation, breast-feeding begins within one hour after birth, and exclusive breast-feeding can last until the baby is 6 months old (5). The oral microbiota of breastfed infants is highly similar to that of their mother's mouth, breast milk and areolas. *Streptococcus* dominates the oral microbiota of exclusively breastfed infants, while *Actinomyces* and *Prevotella* are higher in formula-fed infants (80). *Bifidobacterium* were less represented in the intestinal microbiota of infants fed formula that was not supplemented with probiotics or human milk oligosaccharides (HMO) compared to breastfed children, and supplementation of formula with *Bifidobacterium* did not significantly increase *Bifidobacterium* in the infant gut (81). Differences in the initial microbiota due to different feeding patterns can have long-term effects on the oral and intestinal microbiota of infants (82).

The development and maturation of the innate and adaptive immune systems of neonates is time-dependent. In the first few days, immune-related substances such as secretory immunoglobulins in breast milk are the main source of antibodies and immune cells in newborns (82, 83). Breast milk contains high levels of sIgA, which has a crucial role in pathogen clearance, microbiota colonization and in microbiota homeostasis by influencing microbiota gene expression. Studies have shown that maternal sIgA may regulate the development of the oral microbiota by limiting the colonization of potentially pathogenic species, protecting and regulating the homeostasis of mucosal epithelial cells, and has been found to inhibit the local adhesion of specific pathogenic bacteria (84, 85). SIgA in the feces of breastfed infants around 6 months of age was significantly higher than that of formula-fed infants (86). Breast milk also provides immunoglobulins, complement proteins, lysozyme and lactoferrin, antimicrobial substances that protect infants from pathogens and influence their immune maturation. Depending on the Fc receptor (FcRn) of newborn mice, IgG derived from breast milk can enter the blood stream of mice, which is very important to protect against mucosal diseases induced by *Escherichia coli* (87). The main cytokine present in human milk is IL-10, which suppresses the immune response and participates in tolerance to dietary and microbial antigens (88). Breast milk contains macrophages, neutrophils and lymphocytes, which can be transferred directly to the infant through breast milk and trigger an immune response in the infant and influence the phenotype of the infant's immune cells, particularly the B and T cell phenotype (89, 90).

There are other bioactive components present in human milk, mainly composed of species involved in HMO metabolism in human milk (91, 92). Charbonneau et al. dissected the presence of microbiota-dependent effects of HMO (93). Because HMO can't be digested by infants, they play a role as prebiotics, supporting the growth of some beneficial bacterial strains such as *Bifidobacterium*, *Streptococcus*, *Staphylococcus* and so on in infants' gastrointestinal tract (94), regulates intestinal epithelial cell response and improves intestinal barrier function, provides the primary substrate that shapes the intestinal microbiota of infants and influences the maturation of the intestinal mucosal immune systemand as immunomodulators that compete with potentially pathogenic microbiota to prevent infection and the adhesion and invasion of certain pathogens (95, 96). In addition, HMOs can be fermented by certain gut bacteria to produce short-chain fatty acids(SCFAs), SCFAs are known to be immunomodulators and can even shape the adult immune system by activating G-protein coupled receptor 41/43(GPR 41/43). This pathway was shown to reduce the severity of allergic asthma and colitis in mouse models (97–99). The above studies suggest that increasing HMO content and thus short-chain fatty acid levels by breastfeeding newborns may be a new option for preventing allergic diseases in children.

The above results indicate that breast milk contributes to the establishment of oral and intestinal microbiota, and that immune-related substances in breast milk directly or indirectly promote the development of the infant's immune system. In the long run, breastfeeding is associated with enhanced cognitive development and reduced risk of diseases such as nephrotic syndrome, obesity and type 2 diabetes in children (100–103). However, Gámez-Valdez et al. found that in colostrum samples from women with gestational diabetes mellitus, the GDM group showed higher microbial diversity (104). Also in Piñeiro-Salvador et al. peripheral blood and colostrum paired samples from mothers with BMI > 30 and BMI < 25 were analysed and found that compared to the lean group, the colostrum B lymphocyte compartment was significantly reduced, and CD16 blood monocytes had an increased CD16 expression compared to the lean group in obesity (105). In conclusion, gestational diabetes mellitus (GDM) and obesity affect colostrum composition and lead to abnormal oral and intestinal microbiota colonisation and impact on the establishment of the immune system via the breast milk microbiota and maternal-derived cytokines and leukocytes. Therefore, more long-term follow-up clinical studies are needed to elucidate the influence of maternal factors on breast milk composition and the control mechanisms linking breast milk composition to a diverse infant microbiome and health or disease status later in life.

### 3.3 Effects of antibiotic exposure on infants

Exposure to antibiotics in early life will destroy most oral and intestinal microbiota, reduce the diversity of microbiota and change the bacterial community structure (Table 2). Dzidic et al's research shows that there are a lot of unique bacteria in infants treated with amoxicillin and penicillin in early life (Effect of caries removal status and time, *p* = 0.05).For example, the genus Granulicatella was higher in abundance at 24 months of age (*p* = 0.003) in children not taking antibiotics while Prevotella (*p* = 0.020) was more prevalent at 7 years of age in children treated with antibiotics in early life (82). Kennedy et al. showed that antibiotic treatment was positively correlated with two OTUs in *Pasteurellaceae* and *Neisseriaceae families*, and negatively correlated with OTUs within the Prevotellaceae family (112). It is reported that antibiotic exposure reduces α diversity and increases the abundance of *Enterobacteriaceae*, and antibiotic-specific enrichment of antibiotic resistance genes (ARGs) and multidrug resistant organisms (MDROs) (113, 114).

**Table 2:** Effects of antibiotic exposure on infant microbiota.

Nation	Research type	Sample size	Route of antibiotic use	Type of antibiotic	Changes of microbiota	Reference
Netherlands	Longitudinal study, multi-center	98 infants	intravenous injection	antibiotics	*Bacteroidetes* abundance and diversity were significantly lower at all time points in antibiotic-treated infants compared to controls (*p* = 0.03, *p* = 0.003 and *p* < 0.001 for abundance, and *p* = 0.009, 0.004, 0.004 for diversity, at *T* = 1, *T* = 2 and *T* = 3 respectively	(106)
China	Control study, single-center	9 infants	oral administration + intravenous injection	Cephalosporin, penicillin	The *F/B* ratio decreased by about 1/3 (*p* < .05), with an increase in *Bacteroidetes* and decrease in *Firmicutes*	(107)
Irish	Control study, single-center	18 infants	Parenteral therapy	Ampicillin, gentamicin	Lower levels of *Bifidobacteria* at 4 weeks compared with controls (5% vs. 25%; *p* = .013); lower levels of *Lactobacilli* (1% vs. 4%; *p* < .009)	(108)
Suomi	Longitudinal study, single-center	142 infants	oral administration	macrolide	In the last 6 months, *Proteobacteria* (1.96-fold change *p* < .02), *Clostridium* (2.68-fold change *p* < .004) and *Bacteroides* (2.04-fold change *p* < .004) increased, *Bifidobacterium*(0.23-fold change *p* < .004) and *Lactobacillus* (0.12-fold change *p* < .004) decreased	(109)
Danish	Control study, single-center	72 infants	oral administration	azithromycin	Short-term, azithromycin caused a 23% reduction in observed richness and 13% reduction in Shannon diversity.	(110)
Chile	Longitudinal study, single-center	31 infants	oral administration	amoxicillin	Total *Bifidobacterium* concentrations not significantly altered but complete disappearance of *Bifidobacterium* adolescentis species (0% vs. 36.4%, *p* < .001)	(111)

Antibiotic use may have potential long-term effects on microbiota development. The results of the study showed that the proportion of *Neisseria* and *Streptococcus mitis/dentisani* was increased in the oral cavity of 7-year-old children who had not been exposed to antibiotics in the first two years of life, while the levels of *Prevotella* and *Actinomyces* were higher in children exposed to antibiotics (82). In a study by Yassour et al, antibiotic treatment in the first year of life was found to be associated with a reduction in microbial diversity at 3 years of age (115). At the same time, mouse pups were treated with antibiotics, and it was found that the levels of interleukin IL-4 and IgE increased, the number of Treg cells decreased, and the Treg/Th balance was disrupted (116, 117). Early antibiotic exposure has been reported to predispose to microecological dysregulation and immune system dysfunction, increasing susceptibility to asthma, allergic diseases, IBD, Crohn's disease, type 1 diabetes, and other diseases (118, 119).

### 3.4 Effects of environmental exposure on infants

The results of many studies suggest that it is environmental factors, rather than host genetic factors, that shape the human gut microbiota (120). The biodiversity of the environment, the human commensal microbiota and the human immune system are a complex system of interactions. Current findings show that air pollutants can be ingested directly into the body through the oral cavity with food and liquids, and also by being inhaled into the lungs, where smaller particles reach the alveolar space and are transported by alveolar macrophages to the oropharynx and gastrointestinal tract.The intake of particulate matter will change the intestinal microbiota (for example, the relative numbers of *Bacteroides*, *Firmicutes* and *Verrucomicrons* have changed significantly), or increase the production of ROS and the release of inflammatory factors to promote the disorder of intestinal microbiota (121, 122). Lehtimäki et al. showed that urbanization-related changes in the infant microbiota increase the risk of asthma and atopic features, possibly through crosstalk with the developing immune system (123). In contrast, exposure to the farm environmental microbiota directs the infant gut microbiota toward appropriate tolerances, thereby reducing their risk of developing asthma. Thus, postnatal environmental exposure is an important factor in the infant oral and gut microbiota (124).

## 4 Discussion

Dysbiosis of the oral microbiota increases the risk of developing oral and systemic diseases, and many related diseases overlap with systemic diseases caused by dysbiosis of the gut microbiota (125). Research has shown that the oral microbiota is an endogenous reservoir of the gut microbiota (126). Interactions between multiple factors such as bacterial translocation, circulating bacteria, bacterial metabolites, immune cells, and inflammatory factors influence the homeostasis of the oral and intestinal microbiota in a bidirectional manner (127). Thus, researchers propose that oral microbes are major contributors to the overall health of the host and that the oral-gut axis may serve as a potential causal mechanism linking systemic diseases (128). It is particularly important to establish healthy oral and gut microbiota during the critical window of 1,000 days of life.

The abundance of bifidobacteria in infants decreases with generations and there is a trend toward increased oral and intestinal pathogens due to antibiotic use, the presence of latent pathogens in the mother and the environment, and genetic disorders (129). The depletion and exhaustion of the collective microbial reservoir by the misuse of antibiotics leads to the emergence and spread of drug-resistant microorganisms, making the re-establishment of a healthy microbiota difficult, with increased susceptibility to infections, greater symptomatology and higher mortality rates (130). The World Health Organization has identified antibiotic resistance as one of the top ten global health threats (131). Probiotics have great potential as a “new age” immunotherapy and an alternative to antibiotics. Probiotics are microorganisms that contain sufficient quantities of defined microorganisms to interfere with the growth of pathogenic bacteria through the secretion of bacteriostatic substances, competition with pathogenic bacteria for nutrients and adhesion sites. Enomoto et al. reported that prenatal supplementation of pregnant women with Bifidobacterium breve M-16 V and Bifidobacterium longum BB536 and subsequent postnatal supplementation of newborns may activate the anti-allergic mechanisms of the immature immune system and significantly reduced the risk of eczema/atopic dermatitis found in infants in the probiotic group during the first 18 months of life [OR: 0.231 [95% CI: 0.084–0.628 ] and 0.304 [0.105–0.892] at 10 and 18 months of age, respectively]. In conclusion, probiotics increase the diversity of the oral and intestinal microbiota, enhance immunity in infants and children, and combat viral respiratory infections through strain-specific immunomodulatory effects and stimulation of the interferon (IFN) pathway (132, 133). Numerous basic and clinical studies have demonstrated the effectiveness of probiotics in the treatment of oral and intestinal diseases in infants (Table 3). In addition, probiotics have specific effects on reducing the expression of resistance genes in addition to their general effects on pathogenic bacteria. Studies have shown that probiotic strains such as *C. butyricum MIYAIRI 588* reduce the expression of resistance genes (143). Probiotic strains may be a potential solution for mitigating the problem of antibiotic resistance, and more research is expected to advance the understanding of probiotic strains for mitigating antibiotic resistance.

**Table 3:** Effects of various probiotics on the microbiology of the infant's mouth and intestines.

Probiotics	Research findings	Reference
*Oropharyngeal Probiotic ENT-K12*	Effective in reducing acute and RRTi episodes in school-aged children, shortening the duration of respiratory symptom episodes, reducing the use of antibiotics and antiviral medication, and reducing the number of days children are absent from school and parental work	(134)
*Bifidobacterium lactis* *Probio-M8*	Reducing the duration and frequency of acute RTI symptoms, antibiotic prescribing, and length of stay in hospitalised children under two years of age	(135)
* Lactobacillus rhamnosus* *GG ATCC 53103*	Attenuating microbiota changes in preterm infants by accelerating the *Bifidobacteria*-dominated gut microbiota	(136)
*Lactobacillus paracasei* *N1115*	Enhancement of lactic acid bacteria, increase in faecal sIgA levels maintenance of faecal pH	(137)
* Lactobacillus reuteri* *DSM 17938*	Significant improvement in bowel frequency in functional constipation	(138)
*Lactobacillus paracasei* *CBA L74*	Reducing the incidence of respiratory and gastrointestinal infections in young children attending school	(139)
* Bifidobacterium longum* *subsp.* *infantis* *M-63*	Decreased faecal pH, increased levels of acetic acid and IgA in faeces, and decreased frequency of bowel movements and watery stools. Contributes to the development of a *Bifidobacteria*-dominated gut microbiota at key developmental stages in full-term infants	(140)
*Lactobacillus acidophilus,* *Bifidobacterium bifidum* *and* *Bifidobacterium infantis*	Prevention of NEC and feeding intolerance in preterm babies	(141)
* Lactobacillus rhamnosus* *GG*	The resulting increase in the abundance of *Prevotella*, *Lactococcus* and *Ruminalococcus* and the decrease in *Escherichia coli* appear to prevent penicillin-induced alterations in the microbiota	(142)

It has been found that during the critical window of the first 1,000 days of life, it is possible to regulate the health of newborns in a non-invasive and inexpensive way, such as supplementing with probiotics and prebiotics to regulate and restore the oral and intestinal microbiota in order to reduce children's risk of diseases such as asthma, allergies, and obesity, as well as global morbidity and mortality rates associated with childhood malnutrition (144–147). In the future, personalized infant diets should be further explored and developed to restore microbiota disorders caused by factors such as cesarean section, prenatal or postnatal antibiotics. The study by Gámez-Valdez and Piñeiro-Salvador found that differences in bacterial communities and leukocytes in samples from colostrum from obese women may have an impact on the establishment of an infant's oral and intestinal microbiomes and immune system, and may even pose a threat to the infant's future health (104, 105). Korpela et al. transferred fecal microbiota from mothers to infants to correct gut microbiota imbalances common in CS-born infants and showed that FMT restored microbiota development in infants born vaginally, while FMT attenuated increased levels of potentially opportunistic pathogens in infants born as a result of CS (148). Microbial Ecosystem Transplantation (MET), on the other hand, is a more advanced and precise method than Fecal Microbiota Transplantation (FMT), which involves the purification and cultivation of selected beneficial bacteria from a sample to produce a well-defined and stable microbial ecosystem that can be transplanted into a recipient (149). Therefore, an innovative application of MET may be to address the specific needs of the infant gut microbiota by developing age-specific formulas for the first 1,000 days of life to promote healthy development and prevent or treat dysbiosis. These age-specific MET formulations may have long-term benefits for infant health by supporting immune and metabolic development. Further research is needed to evaluate the safety, efficacy, and long-term consequences of probiotics, FMT, and MET in infants as promising avenues for future research in the field of infant microbiota therapy.Currently, many questions remain about microbiota-immune system interactions, the oro-intestinal axis, or the mechanisms of their interactions with systemic diseases. There is a need for more in-depth exploration of the impact of maternal and child factors (e.g., obesity, diabetes) on the establishment of the microbiota and the immune system, and the mechanism of action of how alterations in the microbiota can increase susceptibility to disease, in order to provide a scientific basis for targeted interventions and therapeutic approaches.
